# Integration of peroxisomes into an endomembrane system that governs cellular aging

**DOI:** 10.3389/fphys.2012.00283

**Published:** 2012-07-17

**Authors:** Adam Beach, Michelle T. Burstein, Vincent R. Richard, Anna Leonov, Sean Levy, Vladimir I. Titorenko

**Affiliations:** Department of Biology, Concordia University, MontrealPQ, Canada

**Keywords:** peroxisome, cellular aging, interorganellar communication, sirtuins, senescence factors, organelle inheritance, proteostasis, autophagy

## Abstract

The peroxisome is an organelle that has long been known for its essential roles in oxidation of fatty acids, maintenance of reactive oxygen species (ROS) homeostasis and anaplerotic replenishment of tricarboxylic acid (TCA) cycle intermediates destined for mitochondria. Growing evidence supports the view that these peroxisome-confined metabolic processes play an essential role in defining the replicative and chronological age of a eukaryotic cell. Much progress has recently been made in defining molecular mechanisms that link cellular aging to fatty acid oxidation, ROS turnover, and anaplerotic metabolism in peroxisomes. Emergent studies have revealed that these organelles not only house longevity-defining metabolic reactions but can also regulate cellular aging via their dynamic communication with other cellular compartments. Peroxisomes communicate with other organelles by establishing extensive physical contact with lipid bodies, maintaining an endoplasmic reticulum (ER) to peroxisome connectivity system, exchanging certain metabolites, and being involved in the bidirectional flow of some of their protein and lipid constituents. The scope of this review is to summarize the evidence that peroxisomes are dynamically integrated into an endomembrane system that governs cellular aging. We discuss recent progress in understanding how communications between peroxisomes and other cellular compartments within this system influence the development of a pro- or anti-aging cellular pattern. We also propose a model for the integration of peroxisomes into the endomembrane system governing cellular aging and critically evaluate several molecular mechanisms underlying such integration.

## Introduction

A growing body of evidence implies that, in addition to the well known roles of the peroxisome in housing fatty acid oxidation and maintaining hydrogen peroxide homeostasis (Poirier et al., 2006; Wanders and Waterham, 2006; Schlüter et al., 2010), this organelle is actively involved in organizing the processes of development, differentiation, and morphogenesis in evolutionarily distant organisms. In mammalian and plant cells, the rate of fatty acid metabolism and the efficiency of reactive oxygen species (ROS) and reactive nitrogen species (RNS) turnover within the peroxisome define the dynamics of changes in the levels of signaling lipids, ROS, and RNS outside this organelle (Desvergne and Wahli, 1999; Corpas et al., 2001; del Río et al., 2006; Nyathi and Baker, 2006). Following their release from the peroxisome, these signaling molecules bind and activate a distinct set of transcription factors that respond by causing global changes in gene expression to initiate certain developmental and differentiation programs (Kersten et al., 2000; Desikan et al., 2001; Hu et al., 2002; Ma et al., 2002; Michalik et al., 2002; Baker et al., 2006; del Río et al., 2006; Michalik and Wahli, 2006; Nyathi and Baker, 2006; Bonekamp et al., 2009; Antonenkov et al., 2010; Ivashchenko et al., 2011; Li et al., 2011; Neher et al., 2012). Thus, the peroxisome functions as an intracellular signaling compartment that can orchestrate important developmental decisions from inside the cell by modulating the extra-peroxisomal concentrations of several potent cellular messengers (Titorenko and Rachubinski, 2004; Terlecky and Titorenko, 2009; Thoms et al., 2009; Dixit et al., 2010). Furthermore, the peroxisome can operate as an organizing platform for several developmental and differentiation programs by compartmentalizing the initial steps of plasmalogen biosynthesis in mammalian and nematode cells, providing acetyl-CoA for the biosynthesis of melanin and glycerol in fungal cells, and carrying out the oxidative decomposition of very long-chain fatty acids, phytanic acid, and pristanic acid in mammalian cells (Powers and Moser, 1998; Motley et al., 2000; Thines et al., 2000; Gould et al., 2001; Kimura et al., 2001; Petriv et al., 2002; Wang et al., 2005; Asakura et al., 2006; Terlecky and Titorenko, 2009; Imazaki et al., 2010; Van Veldhoven, 2010; Goh et al., 2011; Mast et al., 2011; Bhadauria et al., 2012). Moreover, while the peroxisome-associated pools of several bifunctional proteins with dual subcellular localization operate in peroxisome biogenesis and function, their pools in other organelles organize certain processes of development, differentiation, and morphogenesis in mammalian, plant, and yeast cells (Titorenko et al., 1997; Titorenko and Rachubinski, 1998, 2004; Lin et al., 1999; Footitt et al., 2002; Gavva et al., 2002; Geuze et al., 2003; Lin et al., 2004; Slabas et al., 2004; Karnik and Trelease, 2005; Ashibe et al., 2007; Freitag et al., 2012). In addition, the peroxisome provides a template for the formation of the Woronin body, a specialized subcellular compartment that in the filamentous fungi *Neurospora crassa* and *Aspergillus oryzae* is essential for a multistep process in cell morphogenesis initiated by physical damage to hyphae (Jedd and Chua, 2000; Tenney et al., 2000; Liu et al., 2008; Escaño et al., 2009; Jedd, 2011; Liu et al., 2011). In human cells, the peroxisome can also serve as an intracellular platform for the development of the human immunodeficiency virus and rotavirus (Cohen et al., 2000; Mohan et al., 2002).

Recent findings have broadened a spectrum of complex biological processes that depend on the functional integrity of the peroxisome. Emergent evidence supports the view that such peroxisome-confined metabolic processes as fatty acid oxidation, ROS turnover, and anaplerotic replenishment of tricarboxylic acid (TCA) cycle intermediates play essential roles in defining the replicative and chronological age of a eukaryotic cell (Titorenko and Terlecky, 2011). Peroxisomal fatty acid oxidation has been shown to regulate cellular aging because it operates as a system controller that modulates levels of non-esterified fatty acids and diacylglycerol by governing lipid dynamics in peroxisomes, lipid bodies, and the endoplasmic reticulum (ER) (Goldberg et al., 2009a,b; Titorenko and Terlecky, 2011); non-esterified fatty acids are known to accelerate the age-related necrotic and apoptotic cell death mechanisms, whereas the diacylglycerol-activated protein kinase C signaling sensitizes cells to age-related stresses (Spitaler and Cantrell, 2004; Low et al., 2005; Feng et al., 2007; Aksam et al., 2008; Jungwirth et al., 2008). Furthermore, peroxisomal fatty acid oxidation and anaplerotic reactions have been demonstrated to delay cellular aging by potentiating the mitochondrial retrograde (RTG) signaling pathway of longevity regulation (Chelstowska and Butow, 1995; Kos et al., 1995; Epstein et al., 2001; Traven et al., 2001; Jazwinski, 2005b; Liu and Butow, 2006; Titorenko and Terlecky, 2011; Jazwinski, 2012). Moreover, ROS homeostasis and the extent of macromolecular oxidative damage within the peroxisome govern several anti-aging processes confined to this organelle (Morita et al., 2000; Legakis et al., 2002; Aksam et al., 2007; Koepke et al., 2008; Aksam et al., 2009; Lingard et al., 2009; Mathur, 2009; Sinclair et al., 2009; Titorenko and Terlecky, 2011).

The peroxisome defines the replicative and chronological age of a eukaryotic cell not only by operating as a system controller that modulates levels of non-esterified fatty acids and diacylglycerol, replenishes TCA cycle intermediates destined for mitochondria, and contributes to the maintenance of peroxisomal ROS homeostasis and macromolecular oxidative damage. Recent studies have revealed that this organelle can also regulate cellular aging via its communication with other cellular compartments. This dynamic communication involves the establ ishment of extensive physical contact between peroxisomes and lipid bodies, maintenance of an ER to peroxisome connectivity system, exchange of certain metabolites between peroxisomes and other cellular compartments, and bidirectional flow of some protein and lipid constituents between peroxisomes and other organelles. In this review we summarize the evidence that peroxisomes are dynamically integrated into an endomembrane system that governs cellular aging. We discuss various strategies through which peroxisomes are integrated into this endomembrane system, critically evaluate the molecular mechanisms underlying each of these strategies, and analyze the age-related dynamics of communications between peroxisomes and other cellular compartments composing the longevity-defining endomembrane system. We also outline recent progress in understanding how communications between peroxisomes and other cellular compartments within this system influence the development of a pro- or anti-aging cellular pattern. Based on the available evidence, we propose a model for the integration of peroxisomes into the endomembrane system governing cellular aging.

## A role for cytosol-to-peroxisome targeting of Pnc1p in regulating yeast longevity

A support for a distinctive mechanism that underlies the essential role of peroxisomes in regulating cellular aging comes from the observation that Pnc1p, a pyrazinamidase/nicotinamidase 1 that converts nicotinamide to nicotinic acid in the NAD^+^ salvage pathway (Ghislain et al., 2002), is targeted from the cytosol to the peroxisome in response to CR and various mild stresses (Anderson et al., 2003). CR and all of these other “hormetic” stimuli—the term “hormesis” refers to a beneficial defense response of an organism to a low-intensity biological stress (Gems and Partridge, 2008; Rattan, 2008; Calabrese et al., 2011, 2012)—increase the lifespan of replicatively aging yeast in a Pnc1p-dependent manner (Anderson et al., 2003). Peroxisomal import of Pnc1p under conditions of such longevity-extending hormesis requires the peroxisomal targeting signal 2 (PTS2) shuttling receptor Pex7p and the peroxin Pex6p, but does not rely on the PTS1 receptor Pex5p (Anderson et al., 2003). Such specific peroxisomal targeting of Pnc1p, one of the key regulators of replicative aging in yeast (Lin and Sinclair, 2008), in response to their exposure to various anti-aging exogenous factors suggests that Pnc1p in the peroxisome could modulate some longevity-related processes confined to this organelle. What are these processes?

**Figure 1:**
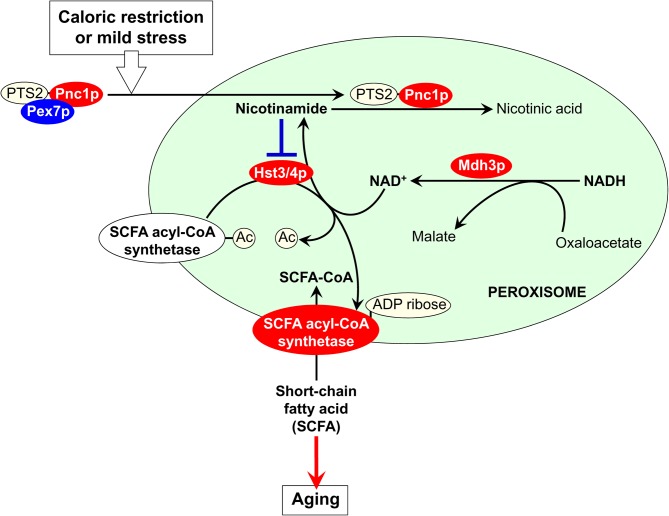
**A proposed role for cytosol-to-peroxisome targeting of Pnc1p in regulating longevity of replicatively aging yeast.** If exposed to caloric restriction (CR) and other hormetic anti-aging stimuli, yeast cells respond by targeting Pnc1p—a pyrazinamidase/nicotinamidase 1 that converts nicotinamide to nicotinic acid in the NAD^+^ salvage pathway—not only to the nucleus but also to the peroxisome. The delivery of Pnc1p to the peroxisome depends on the peroxisomal targeting signal 2 (PTS2) shuttling receptor Pex7p. Inside the peroxisome, Pnc1p activates the sirtuins Hst3p and Hst4p by reducing the concentration of their non-competitive inhibitor nicotinamide. Using NAD^+^ generated by the peroxisomal malate dehydrogenase Mdh3p as a co-substrate in protein deacetylation and ADP-ribosylation reactions, the activated Hst3p and Hst4p stimulate acyl-CoA synthetases required for peroxisomal transport and oxidation of short-chain fatty acids (SCFA). By reducing the levels of these fatty acids in the cytosol, peroxisomes contribute to the beneficial effect of CR and other hormetic stimuli on longevity. See text for details.

The established function of Pnc1p in the nucleus—an organelle to which this protein is also sorted from the cytosol in yeast exposed to CR and other hormetic stimuli (Anderson et al., 2003)—provides a useful hint on the nature of peroxisome-confined processes that could be modulated by Pnc1p under these longevity-extending conditions. In the nucleus, Pnc1p depletes the level of nicotinamide, a strong non-competitive inhibitor of the NAD^+^-dependent protein deacetylase Sir2p required for lifespan extension in yeast under CR conditions (Bitterman et al., 2002). The resulting Pnc1p-driven activation of Sir2p delays replicative aging by suppressing recombination at the ribosomal DNA (rDNA) locus, thereby decreasing the efficiency of extrachromosomal rDNA circle (ERC) formation in the nucleolus (Lin and Sinclair, 2008). It should be stressed that two of the four members of the Sir2p family of NAD^+^-dependent protein deacetylases (*i.e*., sirtuins) in yeast—called Hst3p and Hst4p for being Homologs of SIR Two proteins—drive the metabolism of fatty acids by activating acyl-CoA synthetases for their short-chain species (Starai et al., 2003). By converting short-chain fatty acids into their corresponding acyl-CoA forms, acyl-CoA synthetases enable their cellular and intracellular transport and metabolism (Starai et al., 2003). It has been proposed that both Hst3p and Hst4p activate these acyl-CoA synthetases by deacetylating them and cleaving NAD^+^ in each reaction cycle (Starai et al., 2003). Because sirtuins are also known for their NAD^+^-dependent ADP-ribosylation activity (Haigis and Guarente, 2006; Haigis and Sinclair, 2010), a possibility that Hst3p and Hst4p activate acyl-CoA synthetases for short-chain fatty acids in ADP-ribosylation reactions is also feasible. Altogether, these findings suggest the following hypothesis for a role of cytosol-to-peroxisome targeting of Pnc1p in regulating longevity of replicatively aging yeast (Figure 1). In response to their exposure to CR and other hormetic anti-aging stimuli, yeast cells target Pnc1p not only to the nucleus but also to the peroxisome. Following its PTS2- and Pex7p-dependent import into the peroxisome, Pnc1p depletes the level of nicotinamide, a strong non-competitive inhibitor of Hst3p and Hst4p. As a co-substrate in protein deacetylation and/or ADP-ribosylation reactions, each of these sirtuins could use NAD^+^ known to be generated by the peroxisomal malate dehydrogenase Mdh3p (Kunze et al., 2006). The Pnc1p-dependent depletion of nicotinamide activates Hst3p and Hst4p; in turn, these sirtuins stimulate acyl-CoA synthetases required for peroxisomal transport and oxidation of short-chain fatty acids (Figure 1). We hypothesize that, by depleting the levels of these fatty acids in the cytosol and/or oxidizing them, peroxisomes make an important contribution to the longevity-extending effect of CR and other hormetic stimuli. A critical evaluation of our hypothesis will require testing of the localization of Hst3p and Hst4p to the peroxisome, either permanent or triggered in response to CR and mild stresses. Another key challenge for the future will be to evaluate the ability of peroxisomal acyl-CoA synthetases to undergo reversible deacetylation and/or ADP-ribosylation in an Hst3p- and/or Hst4p-dependent fashion following exposure of yeast to these longevity-extending stimuli.

## The peroxin Pex6p contributes to the maintenance of age asymmetry between the mother and daughter yeast cells with respect to segregation of functional mitochondria

Several peroxisomal proteins are known to possess dual subcellular localization and function (reviewed by Titorenko and Rachubinski, 2004; Mast et al., 2010; Islinger et al., 2012). While the major, peroxisome-bound portion of each of these proteins controls essential processes confined to this organelle, their pools in other organellar compartments govern certain developmental, differentiation, and morphogenetic programs (Titorenko and Rachubinski, 2004; Islinger et al., 2012). The emerged compendium of these bifunctional peroxisomal proteins with dual subcellular localization is on a fast-growing list of the so-called “moonlighting proteins” (Jeffery, 1999, 2011; Shi and Shi, 2004; Kim and Dang, 2005; Gancedo and Flores, 2008; Jeffery, 2009; Flores and Gancedo, 2011). By analyzing the information on dynamic changes in metabolic status and/or organelle functional state within one subcellular location and then moving to other location(s) for initiating an adequate response to such changes, these moonlighting proteins integrate various cellular activities in space and time (Shi and Shi, 2004; Kim and Dang, 2005; Cho et al., 2006; Gancedo and Flores, 2008; Sen et al., 2008; Flores and Gancedo, 2011; Jeffery, 2011).

The peroxin Pex6p is an AAA ATPase (ATPase associated with various cellular activities) whose peroxisome-associated pool has been implicated in peroxisomal protein import (Titorenko and Rachubinski, 2009; Ma et al., 2011; Rucktäschel et al., 2011). In the yeast *Yarrowia lipolytica* Pex6p is a moonlighting protein whose minor portion is confined to the ER (Titorenko et al., 1997; Titorenko and Rachubinski, 1998). The ER-associated Pex6p is an essential component of protein machinery that orchestrates the dimorphic transition from a round yeast form to a filamentous (mycelial) form (Titorenko et al., 1997; Titorenko and Rachubinski, 2004). Pex6p, along with other ER components of this machinery, governs this cell polarization and differentiation program by driving the delivery of mycelium-specific proteins from the ER to the cell surface (Titorenko et al., 1997).

**Figure 2:**
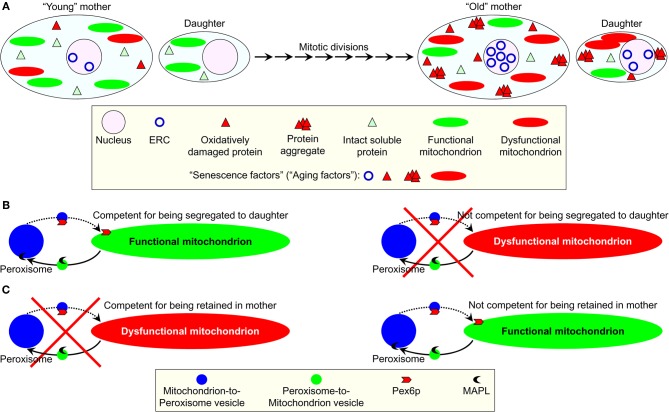
**A proposed role for the mainly peroxisomal protein Pex6p in sequestering dysfunctional mitochondria in the mother cell of replicatively aging yeast and/or segregating functional mitochondria to the daughter cell. (A)** In the reproducing by asymmetric cell division yeast *Saccharomyces cerevisiae*, the budding progeny of replicatively “young” mother cells retains the full replicative capacity by not inheriting such “senescence factors” (also called “aging factors”) as extrachromosomal rDNA circles (ERC), oxidatively damaged proteins, protein aggregates, and dysfunctional mitochondria. In contrast, the daughters of replicatively “old” mother cells inherit these senescence factors. **(B,C)** A peroxisome-associated pool of the peroxin Pex6p has been long known for its essential role in peroxisomal protein import. An “extra-curricular” activity of this protein consists in driving the segregation of functional mitochondria to daughter cells. Pex6p could operate as a “filter” sequestering dysfunctional mitochondria in the mother cell **(B)** and/or segregating functional mitochondria to the daughter cell **(C)**. The mechanism underlying such function of Pex6p may involve a recently discovered vesicular traffic between peroxisomes and mitochondria. See text for details.

Recently, a list of the “extra-curricular” activities of Pex6p has been updated by including to it the essential role that this peroxin plays in regulating yeast longevity. Because the yeast *Saccharomyces cerevisiae* reproduce by asymmetric cell division, replicatively “young” mother cells retain such “senescence factors” (also called “aging factors”) as ERCs, oxidatively damaged proteins, protein aggregates, and dysfunctional mitochondria (Figure 2A) (Jazwinski, 2005a; Erjavec et al., 2007, 2008; Henderson and Gottschling, 2008; Steinkraus et al., 2008; Eldakak et al., 2010; Liu et al., 2010; Zhou et al., 2011). Their budding progeny therefore retains the full replicative capacity by not inheriting ERCs or damaged/aggregated proteins and receiving only functional mitochondria (Henderson and Gottschling, 2008; Erjavec et al., 2008; Steinkraus et al., 2008). In replicatively “old” mother cells, this age asymmetry between the mother and daughter cells is lost. As a result, the daughters inherit all four of the known senescence factors (Figure 2A) (Jazwinski, 2005a; Henderson and Gottschling, 2008; Steinkraus et al., 2008). It should be stressed that the overexpression of Pex6p suppresses the lack of age asymmetry between mother and daughter cells in a strain carrying a point mutation in the nuclear gene *ATP2* encoding the β-subunit of the F_1_ sector of mitochondrial F_0_, F_1_-ATP synthase (Lai et al., 2002; Seo et al., 2007). Moreover, not only Pex6p—along with yet-to-be-identified cytosolic proteins—facilitates the import of Atp2p into mitochondria, but it also drives the segregation of functional mitochondria to daughter cells (Seo et al., 2007). Therefore, it is conceivable that Pex6p could operate as one of the “filters” sequestering dysfunctional mitochondria in the mother cell and/or segregating functional mitochondria to the daughter cell (Figures 2B and 2C). The challenge remains to define the mechanisms underlying the ability of Pex6p to facilitate mitochondrial import of Atp2p and to maintain the age-related asymmetrical segregation of functional mitochondria between mother and daughter cells. Another key challenge for the future will be to establish the mechanism for delivery of Pex6p from peroxisomes to mitochondria. Importantly, not only the biogenesis of these two organelles is governed by common transcriptional pathways, but they also share several key components of their division machineries and are linked through mitochondria-to-peroxisome vesicular traffic (Figures 2B and 2C) (Liu and Butow, 2006; Neuspiel et al., 2008; Andrade-Navarro et al., 2009; Delille et al., 2009; Jazwinski, 2012; Islinger et al., 2012).

## Two mechanisms for preventing the segregation of dysfunctional, oxidatively damaged peroxisomes to the daughter yeast cell during mitosis

Not only peroxisomes in replicatively aging yeast contribute to selective segregation of functional mitochondria to the daughter cell, but they also possess a protein machine that governs their own distribution between mother and daughter cells. Recent studies suggested two mechanisms by which this protein machine may operate in preventing the inheritance of dysfunctional, oxidatively damaged peroxisomes by the daughter cell during mitosis.

In *S. cerevisiae*, the inheritance of peroxisomes by daughter cells relies on the peroxisomal protein Inp2p (Fagarasanu et al., 2006). By acting as a receptor for the class V myosin motor Myo2p, Inp2p tags peroxisomes for their segregation to the daughter cell (Fagarasanu et al., 2009). It is conceivable that such Inp2p-dependent tagging of peroxisomes plays a longevity-extending role by enabling the inheritance of only functional peroxisomes by daughter cells. Importantly, the phosphorylation of Inp2p makes it susceptible to degradation, thereby impairing the segregation of Inp2p-less peroxisomes to the daughter cell (Fagarasanu et al., 2009, 2010). One could therefore speculate that such phosphorylation and degradation target mainly Inp2p on dysfunctional, oxidatively damaged peroxisomes for sequestering them in the mother cell.

In another yeast species, *Y. lipolytica*, the inheritance of only newly formed from the ER template peroxisomes may prevent the segregation of their oxidatively damaged, “old” counterparts to the daughter cell during mitosis (Chang et al., 2009). By possessing a dual role in the formation of new peroxisomes from the ER template and in the recruitment of the class V myosin motor Myo2p to their membranes, the peroxins Pex3p and Pex3Bp may enable the selective segregation of these newly formed peroxisomes to the daughter cell, thereby allowing to retain the entire population of dysfunctional, oxidatively damaged peroxisomes in the mother cell (Chang et al., 2009; Fagarasanu et al., 2010).

The challenge remains to define the molecular mechanisms underlying the proposed selectivity in (1) phosphorylating Inp2p only on dysfunctional, oxidatively damaged peroxisomes; and (2) targeting Myo2p only to the ER-confined pool of Pex3p.

## A model for the integration of peroxisomes into an endomembrane system that governs cellular aging

**Figure 3:**
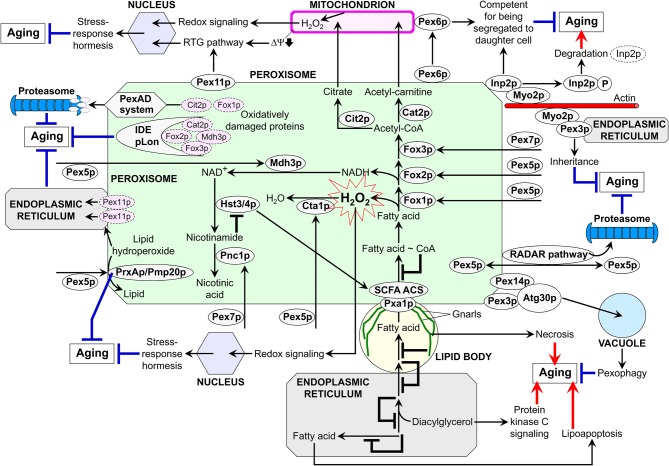
**A model for the dynamic integration of peroxisomes into an endomembrane system governing cellular aging.** Several mechanisms underlie the essential contribution of peroxisomes to the regulation of cellular aging. Each of these mechanisms relies on a network of communications between peroxisomes and other organelles through the maintenance of the endoplasmic reticulum-peroxisome connectivity, establishment of the extensive physical contact with lipid bodies, exchange of certain metabolites, and/or the bidirectional flow of some of their proteins and lipids. Thus, peroxisomes are dynamically integrated into an endomembrane system that governs cellular aging. By modulating the dynamics of communication between peroxisomes and other cellular compartments, the age-dependent efficiency of peroxisomal protein import influences a compendium of longevity regulation pathways relying on such communication. Peroxisomes promote the development of a pro-aging pattern within an endomembrane system governing cellular aging if the overall efficiency of peroxisomal protein import is actively maintained at a sufficiently high level. Conversely, peroxisomes trigger certain pro-aging processes within this endomembrane system if the overall efficiency of peroxisomal protein import is lower than this critical level. See text for details. IDE, insulin degrading enzyme; PexAD, peroxisome-associated protein degradation; pLon, peroxisomal Lon protease; RADAR, receptor accumulation and degradation in the absence of recycling; RTG, retrograde; SCFA ACS, short-chain fatty acid acetyl-CoA synthetase; ΔΨ, electrochemical potential across the inner mitochondrial membrane.

A body of evidence summarized here and elsewhere (Titorenko and Rachubinski, 2004; Titorenko and Terlecky, 2011; Islinger et al., 2012) implies that peroxisomes contribute to the regulation of cellular aging via several different mechanisms. In each of these mechanisms, peroxisomes communicate with other organelles by establishing extensive physical contact with lipid bodies, maintaining the ER-peroxisome connectivity, exchanging certain metabolites, and/or being involved in the bidirectional flow of some of their protein and lipid constituents. Thus, peroxisomes are dynamically integrated into an endomembrane system that governs cellular aging. We propose a model for such integration (Figure 3). The central tenet of this model is that the age-dependent efficiency of protein import into the peroxisome modulates the dynamics of its communication with other cellular compartments, thereby influencing several longevity regulation pathways that rely on such communication. The overall efficiency of peroxisomal protein import is defined by the efficiencies of binding of Pex5p and Pex7p—the PTS1 and PTS2 cytosolic shuttling receptors, respectively—to their cargo proteins in the cytosol, translocation of the receptor-cargo complexes across the peroxisomal membrane, and receptor recycling (Ma et al., 2011; Rucktäschel et al., 2011). Importantly, the efficiencies of all these processes are reduced with age (Legakis et al., 2002; Terlecky et al., 2006; Titorenko and Terlecky, 2011). In our model, if the overall efficiency of protein import into peroxisomes is actively maintained at a sufficiently high level, these organelles trigger certain anti-aging processes within the endomembrane system governing cellular aging (Figure 3). Conversely, if the overall efficiency of peroxisomal protein import is lower than this critical level, peroxisomes promote the development of a pro-aging pattern within this endomembrane system (Figure 3).

Our model envisions that the efficient Pex5p-dependent peroxisomal import of the ROS scavenging enzymes catalase (Cta1p in yeast) and peroxiredoxin (PrxAp in mammals and Pmp20p in yeast) in replicatively and chronologically “young” cells aids in minimizing the oxidative damage to peroxisomal proteins and membrane lipids (Figure 3; Antonenkov et al., 2010; Titorenko and Terlecky, 2011; Ivashchenko et al., 2011). At the surface of the peroxisome, quality control of the Pex5p-driven protein import in these cells is governed by the receptor accumulation and degradation in the absence of recycling (RADAR) pathway for the recycling of Pex5p (Figure 3; Léon et al., 2006; Ma et al., 2011; Titorenko and Terlecky, 2011). Inside the peroxisome, the insulin degrading enzyme (IDE), peroxisomal Lon (pLon) protease, and peroxisome-associated protein degradation (PexAD) system carry out the degradation of oxidatively damaged peroxisomal matrix proteins that are incapable of supporting the anti-aging processes orchestrated by functionally active peroxisomes (Morita et al., 2000; Aksam et al., 2007, 2009; Lingard et al., 2009). A healthy population of such peroxisomes in “young” cells is also sustained by pexophagy, an autophagy-related process in which dysfunctional peroxisomes carrying oxidatively damaged proteins are selectively degraded following their sequestration by vacuoles of yeast and plant cells or lysosomes of mammalian cells (Figure 3; Aksam et al., 2007; Farré et al., 2008; Manjithaya et al., 2010). In plant cells exposed to oxidative stress, the ER-peroxisome connectivity enables the retro-flow of oxidatively damaged matrix proteins as well as of membrane proteins and lipids to the ER—thereby contributing to the maintenance of a healthy population of functionally active peroxisomes (Mathur, 2009; Sinclair et al., 2009).

In our model, peroxisomes in “young” cells not only actively avoid the oxidative damage to their protein and lipid constituents but also operate as a signaling platform that, by maintaining ROS concentration at a certain “optimal” level, delays cellular aging by inducing stress-response hormesis (Figure 3; Titorenko and Terlecky, 2011). At such a level, ROS are unable to elicit substantial oxidative damage to cellular macromolecules but can activate several redox signaling networks known to elevate the abundance and/or activity of stress-protecting and other anti-aging proteins (D'Autréaux and Toledano, 2007; Giorgio et al., 2007; Veal et al., 2007).

Furthermore, the PTS1 and PTS2 cytosolic shuttling receptors Pex5p and Pex7p drive peroxisomal import of Fox1p, Fox2p, and Fox3p (Hiltunen et al., 2003). The efficient import of these core enzymes of fatty acid β-oxidation into peroxisomes of “young” cells increases the efficacy with which they decompose fatty acids derived from triacylglycerols that are synthesized in the ER and deposited within lipid bodies (Goodman, 2008; Goldberg et al., 2009a,b; Kohlwein, 2010). Due to such accelerated peroxisomal fatty acid oxidation and the resulting decrease in the concentrations of non-esterified fatty acids and diacylglycerol, “young” cells escape the premature death by resisting lipid-induced necrosis and apoptosis and by sustaining stress resistance through the attenuation of diacylglycerol-activated protein kinase C signaling (Figure 3; Goldberg et al., 2009a,b; Titorenko and Terlecky, 2011). Another way for the longevity-extending acceleration of peroxisomal fatty acid oxidation in “young” cells is the governed by sirtuins Hst3p and Hst4p stimulation of acyl-CoA synthetases that are required for peroxisomal transport and oxidation of short-chain fatty acids. This anti-aging process is driven by the efficient Pex5p- and Pex7p-dependent peroxisomal import of Mdh3p and Pnc1p for synthesizing a substrate and decomposing an inhibitor of the sirtuins, respectively (Figure 3).

Moreover, the longevity-extending ability of peroxisomes to promote the anti-aging RTG signaling pathway of peroxisomes-mitochondria, mitochondria-nucleus, and nucleus-peroxisomes communications in “young” cells is enhanced by the highly efficient peroxisomal import of Fox1p, Fox2p, Fox3p, Cit2p, and Cat2p in these cells (Figure 3; Titorenko and Terlecky, 2011). Fox1p, Fox2p, and Fox3p are involved in the peroxisomal oxidation of fatty acid to acetyl-CoA following their Pex5p- and Pex7p-dependent delivery to peroxisomes, whereas the citrate synthase Cit2p and acetyl-carnitine synthase Cat2p are imported into these organelles with the help of Pex5p to catalyze the anaplerotic conversion of acetyl-CoA to citrate and acetyl-carnitine (Figure 3; Epstein et al., 2001; Traven et al., 2001; Hiltunen et al., 2003; Titorenko and Terlecky, 2011). The longevity-extending RTG signaling pathway in “young” cells is further amplified through the Pex11p-driven proliferation of peroxisomes and the resulting increase in the effectiveness with which the confined to these organelles fatty acid oxidation and anaplerotic reactions replenish TCA cycle intermediates destined for mitochondria (Figure 3; Jazwinski, 2005b; Liu and Butow, 2006; Titorenko and Terlecky, 2011). It should be emphasized that, by maintaining the functionality of mitochondria in “young” cells, the peroxisome-driven RTG pathway controls the homeostasis of mitochondrial ROS (Titorenko and Terlecky, 2011). This enables the ROS-dependent activation of several redox signaling networks aimed at increasing the levels of stress-protecting and other anti-aging proteins or post-translationally activating some of them (Figure 3; D'Autréaux and Toledano, 2007; Giorgio et al., 2007; Veal et al., 2007). In our model, the segregation of functional mitochondria to the “young” daughter cell and/or the sequestration of dysfunctional mitochondria in the “old” mother cell in replicatively aging yeast are/is driven in part by the delivery of the peroxin Pex6p from peroxisomes to mitochondria through a mechanism that remains to be established (Figure 3; Lai et al., 2002; Seo et al., 2007).

According to our model, *S. cerevisiae* Inp2p—a peroxisome-specific receptor for the class V myosin motor Myo2p—tags peroxisomes for their segregation to the “young” daughter cell in a process that may play a life-extending role by enabling the inheritance of only functional peroxisomes (Figure 3; Fagarasanu et al., 2009, 2010). Furthermore, by possessing a dual role in the formation of new peroxisomes from the ER template and in the recruitment of Myo2p to their membranes, the *Y. lipolytica* peroxins Pex3p and Pex3Bp may enable the inheritance of only newly formed from the ER template peroxisomes thus preventing the segregation of their oxidatively damaged, “old” counterparts to the daughter cell during mitosis (Figure 3; Chang et al., 2009; Fagarasanu et al., 2010).

Our model envisions that the overall efficiency of peroxisomal protein import gradually decreases with replicative and chronological age (Figure 3). A steady, age-related increase in the concentration of peroxisome-confined proteins that are oxidatively damaged by peroxisomally produced ROS could be the driving force for such deterioration of peroxisomal protein import efficiency. The Pex5p-dependent peroxisomal import of catalase—due to the age-dependent decline in the efficiency of its binding to Pex5p and in the extent of Pex5p recycling—is the most sensitive to oxidative damage peroxisomal process (Legakis et al., 2002; Terlecky et al., 2006). The resulting deceleration of catalase import into peroxisomes increases the extent of oxidative damage to their proteins and lipids, thereby initiating the “deterioration spiral” that eventually lowers the overall efficiency of peroxisomal protein import below a critical level. Consequently, the role of peroxisomes in the regulation of cellular aging is switching from being a platform for activating a compendium of anti-aging processes within the endomembrane system governing cellular aging to becoming a platform for the development of a pro-aging pattern within this endomembrane system (Titorenko and Terlecky, 2011). Specifically, the RADAR pathway, IDE and pLon proteases, PexAD system, and pexophagy eventually fail due to the progressive, age-dependent accumulation of oxidatively damaged proteins and lipids in peroxisomes. Thus, cellular aging coincides with the build-up of dysfunctional peroxisomes that are unable anymore to support the anti-aging processes within the endomembrane system governing such aging. Among these impaired anti-aging processes are (1) the peroxisome- and mitochondria-driven pathways of stress response hormesis; (2) the Hst3p/Hst4p-dependent stimulation of acyl-CoA synthetases for peroxisomal transport and oxidation of short-chain fatty acids; (3) the RTG signaling pathway of peroxisomes-mitochondria, mitochondria-nucleus, and nucleus-peroxisomes communications; (4) the Pex6p-dependent sequestration of dysfunctional mitochondria in the “old” mother cell and/or segregation of functional mitochondria to the “young” daughter cell; and (5) the Inp2p-, Pex3p-, and Pex3Bp-dependent segregation of functional peroxisomes to the “young” daughter cell (Figure 3). Moreover, our model foresees that, by being unable to maintain low levels of non-esterified fatty acids and diacylglycerol, the dysfunctional, oxidatively damaged peroxisomes accumulated in aged cells (1) activate the longevity-shortening necrotic and apoptotic cell death mechanisms induced by non-esterified fatty acids; and (2) are unable to attenuate the diacylglycerol-activated protein kinase C signaling that reduces stress resistance (Figure 3).

## Conclusion

Growing evidence supports the view that peroxisomes govern cellular aging via several different mechanisms involving their dynamic communication with other cellular compartments. An important conceptual advance in our understanding of the inherent complexity of cellular aging is that the age-related dynamics of communications between peroxisomes and various other organelles modulates a compendium of longevity regulation pathways. It is conceivable therefore that the peroxisome is dynamically integrated into an endomembrane system governing cellular aging. Much progress has recently been made in defining how communications between peroxisomes and other cellular compartments influence the development of a pro- or anti-aging pattern within this endomembrane system. The challenge remains to define the molecular mechanisms underlying the integration of peroxisomes into the endomembrane system governing cellular aging. Future work will aim at understanding how peroxisomes switch their role in the regulation of cellular aging from being a platform for activating a compendium of anti-aging processes confined to this endomembrane system in “young” cells to becoming a platform for the development of a pro-aging pattern within this endomembrane system in “old” cells. This knowledge will provide greater insight into the mechanisms underlying longevity regulation and is expected to reveal novel targets for anti-aging pharmaceuticals that can extend longevity by modulating the age-related dynamics of communications between peroxisomes and other cellular compartments.

### Conflict of interest statement

The authors declare that the research was conducted in the absence of any commercial or financial relationships that could be construed as a potential conflict of interest.
